# Potential Nutrition and Health Claims in Deastringed Persimmon Fruits (*Diospyros kaki* L.), Variety ‘Rojo Brillante’, PDO ’Ribera del Xúquer’

**DOI:** 10.3390/nu12051397

**Published:** 2020-05-13

**Authors:** Laura Domínguez Díaz, Eva Dorta, Sarita Maher, Patricia Morales, Virginia Fernández-Ruiz, Montaña Cámara, María-Cortes Sánchez-Mata

**Affiliations:** 1 Nutrition and Food Science Department, Pharmacy Faculty, Complutense University of Madrid (UCM), Plaza Ramón y Cajal, s/n, E-28040 Madrid, Spain; ladoming@ucm.es (L.D.D.); edorta@ucm.es (E.D.); patricia.morales@farm.ucm.es (P.M.); vfernand@farm.ucm.es (V.F.-R.); mcamara@farm.ucm.es (M.C.); 2 Canarian Institute of Agrarian Research, E-38270 Tenerife, Spain; 3 School of Pharmacy, University College London, Bloomsbury, London WC1N 1AX, UK; sarita.maher.15@ucl.ac.uk

**Keywords:** persimmon, *Diospyros kaki*, Rojo Brillante, bioactive compound, nutrition claim, health claim

## Abstract

In Europe, nutrition and health claims made on food must be based on scientific evidence, which means a comprehensive evaluation by the European Food Safety Authority (EFSA) prior to authorisation. Processed foods are subject to numerous claims derived from the presence of bioactive compounds; however, natural food products, often the original sources of those compounds, are not habitually the subject of these claims. Although the consumption of persimmon fruit has important health benefits, up to date no specific health claims are authorised for this fruit. In this work, ‘Rojo Brillante’ persimmon fruits (*Diospyros kaki* L.), Protected Designation of Origin (PDO) ‘Ribera del Xúquer’ were characterized regarding the presence of fiber (soluble and insoluble), vitamin C (ascorbic and dehydroascorbic acids), carotenoids (neoxanthin, violaxanthin, β-cryptoxanthin, lycopene, β- carotene) and mineral elements (Fe, Cu, Zn, Mn, Ca, Mg, Na, K). Different fruit batches harvested in different seasons were analyzed by standardized analytical methods (Association of Official Analytical Chemists, AOAC), high-performance liquid chromatography with ultraviolet detection (HPLC-UV) and atomic absorption spectroscopy. Based on the results, Persimon^®^ is potentially able to show two nutrition claims “Source of fiber” and “Sodium-free/salt-free”. This work could set the ground for future studies and to start considering natural food products as candidates for the use of approved claims.

## 1. Introduction

Persimmon fruit (*Diospyros kaki* L.), also known as ‘Sharon fruit’, ‘Caqui’, ‘Kaki’, and ‘Japanese persimmon’, is a fleshy and fibrous tropical fruit that belongs to the *Ebenaceae* family. Although native to China, Taiwan and Myanmar, the cultivation of persimmon fruits has strongly increased in new areas of the world such as the Mediterranean region. The greatest worldwide persimmon producers are China (3.93 million tons, constituting 72.3% of the global production), South Korea (405.70 kt) and Spain (311.40 kt). In addition, Spain is a major exporter of the fruit, particularly within Europe, being ranked in the top 10 of the global exports [1,2,3].

**Figure 1:**
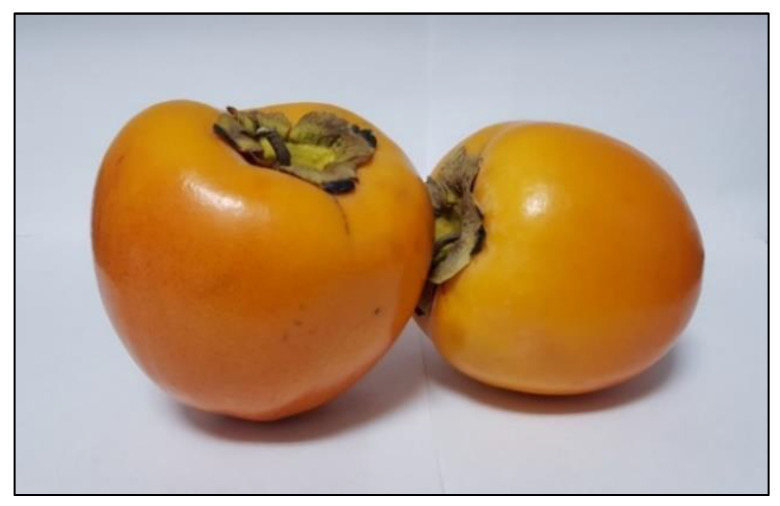
Rojo Brillante persimmon fruits (*Diospyros kaki* L.), Protected Designation of Origin (PDO) ‘Ribera del Xúquer’ samples analyzed in the present study.

In northern hemisphere countries, as in the case of Spain, persimmon fruit is generally harvested from late September to early January and it is normally marketed when the fruit has little acidity and primarily a sweet flavor. The persimmon’s physical attributes include its orange colored pulp and the absence of seeds, which are attractive characteristics of these fruits. There are varieties that show a high astringency, which can be considered an undesirable characteristic. This is due to the chemical nature of tannins, being insoluble in non-astringent varieties (‘Fuyu’, ‘Hana-Fuyu’, ‘Imoto’, ‘Izu’, ‘Jiro’, ‘Okugosho’, ‘Suruga’, etc.) and soluble in the astringent ones (‘Rojo brillante’, ‘Hachiya’, ‘Homan Red’, ‘Ormond’, ‘Tamopan’, ‘Tanenashi’, ‘Taubata’, ‘Triumph’…). Soluble tannins can link to salivary proteins and cause their precipitation or aggregation, giving rise to a rough ‘sandpapery’ or dry sensation in the mouth [4]. ‘Rojo Brillante’ constitutes one of the most important commercial varieties of persimmon in Spain. It is a pointed shape astringent variety (Figure 1) spontaneously originated and produced in the East region of Spain, along the rivers Xúquer and Magro, at the lowlands of the valley, with compact and rich soils, and a mild climate [4,5]. These fruits are protected under the Protected Designation of Origin (PDO), according to Regulation (EU) No 1151/2012, certified by the Regulatory Council of PDO ‘Kaki Ribera del Xúquer’ and accredited by the Spanish National Accreditation Body (ENAC) which ensures quality and traceability standards along the whole process [6]. Some authors have studied the physiological properties, as well as some components of ‘Rojo Brillante’ persimmon fruits, including soluble sugars, carotenoids and phenolic compounds; however, to the authors’ knowledge, no data about other components such as fiber, vitamin C or minerals in the fruits of this variety have been reported [7,8,9].

Since astringent varieties of persimmon can only be consumed when they are over-ripe, different strategies have been developed to reduce the astringency of persimmon fruits leading to fruits than can be commercialized and consumed keeping their firmness. This can be done by applying technologies such as high hydrostatic pressure, or by storing it in modified atmospheres (CO_2_ or ethanol enrichment) which favors the anaerobic respiration conditions and the precipitation of tannins responsible for astringency perception [10,11,12]. The fruits of ‘Rojo Brillante’ variety, PDO ‘Kaki Ribera del Xúquer’ can be treated in this way, leading to the registered brand Persimon^®^ fruits. Several studies have been conducted on the physiological, sensorial and commercial characteristics of deastringed persimmon fruits [7,8,13]; however, the nutritional composition and bioactive compounds present in treated varieties have been very scarcely studied.

Nutrition and health claims made on food labeling, appearance and/or advertising are tightly regulated within the European Union (EU) [14]. Regulation (EC) No 1924/2006 defines a claim as “any message or representation, which is not mandatory under community or national legislation, including pictorial, graphic or symbolic representation, in any form, which states, suggests or implies that a food has particular characteristics”. Claims are authorized under strict conditions and must be based on strong scientific evidence, which means a specific harmonized evaluation by the European Food Safety Authority (EFSA) prior to its authorization [14,15]. Claims must be clear, accurate and reliable; with the wording being an immensely important factor so that the declared beneficial effects are easily understood by the average consumer, as they strongly influence consumer dietary behavior and food choices [14]. In addition, the food industry must meet the specific use conditions for each claim as well as ensure that (1) these claims are consistent with generally accepted nutrition and health principles and (2) those food products displaying claims have a proper nutritional profile [16,17]. Processed foods are the subject of a great number of nutrition and health claims derived from the presence of bioactive compounds; however, natural food products, being often the original sources of those compounds for the food industry, are not habitually the subjects of these claims.

The aim of the present work is to identify the potential components in deastringed persimmon fruits (variety ‘Rojo Brillante’, PDO ‘Ribera del Xúquer’) that can be subjected to nutrition and/or health claims according to the present European Regulations, in terms of fiber, vitamin C, mineral (macro and microelements) and carotenoids content.

## 2. Materials and Methods

### 2.1. Sampling

For analysis, six batches, a total of 192 units (32 per batch) of persimmon fruits, were received from November to December 2017 (batches K1, K2 and K3) as well as in November 2018 (batches K4, K5 and K6). Approximately 8–11 units from each batch were randomly selected and weighed, and the values were recorded in order to calculate the mean weight. On the same day, fruits were fully peeled leaving the edible pulp of the fruit.

Half of each fruit was homogenized for analysis required on fresh fruits. The other half was sliced and frozen at −20 °C for a minimum of 24 h in order to prepare the dry sample useful for further analysis. Subsequently, a Telstar LyoQuest freeze-dryer was employed for freeze-drying and preservation of the samples. The fruits were kept in the freeze-dryer protected from light, to ensure its conservation, at −50 °C and a pressure of 0.100 mBar for a minimum of 72 h. The dried persimmon fruits were then homogenized with a domestic Vorwerk Thermomix 3300 mixer. The powdered lyophilized samples were stored in airtight containers, in the dark and at a controlled temperature to avoid alteration of the product. This dried sample was destined for the determination of dietary fiber content, mineral composition and carotenoid content.

### 2.2. Analytical Methods

All the analytical determinations were performed in triplicate; with the exception of the total dietary fiber assay samples, which were measured in quadruplicate. In order to control the trueness of data obtained, reagent blanks were prepared regularly and certified reference materials were measured together with each sample.

Moisture content, pH, titratable acidity and degrees Brix (°Brix) were analyzed in homogenized fresh samples using AOAC (Association of Official Analytical Chemists) procedures [18]. AOAC 984.25 procedure was employed to determine moisture content; by desiccation of the sample at 105 °C ± 2 °C for 4–6 h, until a constant weight was identified. A potentiometer MicropH-2000, Crison Instrument, was used in order to measure the pH over a homogenized sample 1/10 (w/v) in distilled water; in accordance with AOAC 981.12 procedure. The equipment was adjusted with buffer solutions of pH 4 and 7 to the working temperature, prior to reading the sample pH. This same sample solution was used to assess titratable acidity (TA) by acid-base titration with 0.1 N NaOH, until a pH of 8.1 was reached. This was in accordance with the AOAC 942.15 procedure. The acidity was expressed as mL of 0.01 N NaOH needed to neutralize the acids present in 100 g of edible portion. Finally, following AOAC 932.14C procedure, Degrees Brix were determined with an ATAGO Digital BRIX PR-1 refractometer. The results were expressed as °Brix, percentage of sucrose measured at 20 °C [19,20].

#### 2.2.1. Fiber Analysis

The determination of dietary fiber (soluble, insoluble and total fiber) was carried out by enzymatic-gravimetric methods according to the official methods AOAC 993.19 and 991.42 [21]. An aliquot of 0.3 g of the dried sample was weighed in a conical flask, and phosphate buffer (0.08 M, pH 6) and α-amylase enzyme (Sigma-Aldrich; A 3306 α-Amylase, heat stable solution) were added. α-amylase is added to break down starch into sugars by hydrolysis of the α-(1-4) glucan links in polysaccharides of three or more α-(1-4) linked D-glucose units, without hydrolyzing the α-(1-6) bond [22]. The mixture was incubated at 100 °C for 15 min in the Memmert water bath, with gentle agitation at 5-min intervals. The samples were cooled to room temperature, and NaOH (0.275 N) was used to adjust the pH until 7.5. Subsequently, protease enzymes (Sigma-Aldrich; P 3910 Protease from *Bacillus licheniformis* lyophilised powder) were added for a second enzymatic treatment. The mixture was then incubated for 30 min at 60 °C. The pH was adjusted to 4–4.6 with HCl (0.325 N), and amyl glucosidase enzyme (Sigma-Aldrich; A 9913 Amyloglucosidase solution from *Aspergillus niger*) was added for the final 30-min incubation. The insoluble fraction was obtained after filtration using a Fisherband FB70155 scientific vacuum. The filtered solution was collected in a 500 mL Erlenmeyer flask and ethanol was added to precipitate the soluble fraction, which was obtained 24 h later by vacuum filtration. The residues of both filtrations were dried at 100 °C in a Memmert Loading Modell 100–800 Beshikung oven, and the ash and protein content were then determined. Ash content was gravimetrically quantified after the incineration of residues at 450 °C in a microwave oven (Muffle Furnace MLS 1200 model, Monroe, USA). On the other hand, protein content was calculated by the determination of the nitrogen following the Kjeldahl method. The residues from the fiber determination were transferred to a Kjeldahl tube, and K_2_SO_4_/CuSO_4_ (catalyst) as well as H_2_SO_4_ N/10 were added. Organic matter was removed by digestion with sulphuric acid into a Büchi Digestion Unit K-435, and then distillation was performed with a Büchi Distillation Unit K-350. Finally, an acid-base titration with NaOH N/10 was carried out in order to determinate the total nitrogen contained in the sample. The total dietary fiber content was calculated through the sum of both insoluble and soluble fractions. The results were expressed as g of fiber (soluble, insoluble and total content)/100 g of fresh weight (fw) [23].

#### 2.2.2. Vitamin C Analysis

Vitamin C can be presented in food in the form of L-ascorbic acid (AA) or in its oxidized form L-ascorbic acid dehydro (DHA) with the same vitamin activity. The determination of total vitamin C content (AA + DHA) in the persimmon samples was carried out by an extraction in an acid medium. Subsequent identification and quantification were completed by reverse-phase high-performance liquid chromatography (HPLC) with ultraviolet (UV) detection, using a previously validated method [24]. An aliquot of 5 g of the homogenized fresh portion was weighed and metaphosphoric acid 4.5% (w/v) was added. The mixture was shaken in a magnetic stirrer (P-Selecta, Asincro) for 15 min and protected from light. The extract was filtered with an Albet paper filter No. 1242. A small aliquot of the extract was filtered again using a 0.45 μm polyvinylidene fluoride membrane filter (Millex) and injected in HPLC for the quantification of AA. The reduction of DHA to AA is necessary, as DHA does not absorb practically in UV. Thus, 5 mL of the acid extract was reserved and the reducing agent L-cysteine was added. The pH was adjusted to 7 by using 20% HK_2_PO_4_ and reduced to pH = 3 with 20% metaphosphoric acid, and was made up to 25 mL with metaphosphoric acid 4.5%. The extract was then filtered with a 0.45 μm PVDF membrane filter (Millex) for subsequent injection into HPLC equipment and determination of total vitamin C content (AA and DHA). DHA content was calculated by difference (total vitamin C minus AA). The chromatographic equipment (Micron Analytical) incorporates an isocratic pump model PU-II, automatic injector AS-1555 (model Jasco), UV–visible detector (Thermo Separation Specta Series UV100) and column ODS Sphereclone (2) (250 × 4, 60; 5 μm). The wavelength, mobile phase and flux used were 245 nm, H_2_SO_4_ (1.8 mM, pH 2.5–2.6) and 0.9 mL/min respectively. The data obtained were analysed with the software Biocrom 2000, 3.0. The results were expressed in mg vitamin C/100 g of edible portion [25].

#### 2.2.3. Carotenoids Analysis

##### Ultrasound-Assisted Extraction (UAE)

The method for carotenoid extraction was adapted from one used by Olives Barba et al. (2006) with slight modifications [26]. The extraction was carried out by ultrasound-assisted extraction (UAE); this method allows cell rupture by cavitation improving mass transfer of extractants [27]. An aliquot of 2 g of the dried sample was placed in a topaz vessel and mixed with 50 mL of the prepared extraction solvent, hexane:acetone:ethanol (50:25:25 v/v/v). The mixture was stirred magnetically for 10 min and then placed in an ultrasonic water bath (Ultrasons-H, Selecta) for 10 min. Later, 5 mL of distilled water was added and kept in darkness for 1 h. Two phases were formed and the entire volume of the organic phase (hexane) was taken. The extract was evaporated to dryness with N_2_ flow under vacuum to remove the organic solvent. The residue was dissolved to a volume of 2 mL with the mixture of tetrahydrofuran (THF):acetonitrile (ACN) (15:85). The final solution was filtered using a 0.45 μm membrane filter and injected for HPLC analysis.

##### Chromatographic Conditions

The HPLC analysis was performed according to the procedure previously described by Olives Barba et al. (2006) with some changes [26]. Chromatographic separation was carried out on a C18 μBondapak (300 mm × 3.9 mm, 10 μm) (Waters, EEUU) column at room temperature. The mobile phase consisted of methanol:ACN (5:95 v/v) in an isocratic mode with 0.9 mL/min flow rate. Separation was carried out over 35 min and the absorbance was obtained at 475 nm. The carotenoid compounds in persimmon fruits were identified by the retention time and through external commercial standards. Concentration was expressed as µg per 100 g fw.

##### Standard Carotenoid Preparation

Neoxanthin (95.0%), β-cryptoxanthin (95.0%), lycopene (95.0%), zeaxanthin (95.0%), β-carotene (95.0%) and violaxanthin (95.0%) standards were acquired from CaroteNature (Lupsingen, Switzerland). Individual stock solutions were prepared every day by adding the required and specific solvent to the vial containing the carotenoid standard and mixing until complete dilution at final concentration of 1 mg/mL. Due to solubility problems of carotenoids, several solvents such as hexane, methanol, ethanol, ACN, THF, dimethyl sulfoxide (DMSO), trichloromethane, ethyl ether and different mixtures of the last one were used to determine which was the optimum for each standard. The solvents used to dissolve the standards are shown in Table 1. The stock solutions were diluted according to the instructions of Table 1 in order to provide a series of standard solutions at appropriate concentrations for obtaining linear calibration curves. The linearity of the method was confirmed by regression statistics. The correlation coefficients and the relative standard deviation (RSD) of the slopes were always >0.991 and <5%, respectively. The intra-day and inter-day reproducibility was achieved by analysing six standards of each carotenoid identified [26]. Vitamin A activity was measured as retinol equivalents (RE), being 1 μg RE = 1 μg of retinol, equivalent to 6 μg of β-carotene or to 12 μg of other carotenoids with provitamin A activity (e.g., α-carotene or β-cryptoxanthin) [28].

#### 2.2.4. Mineral Composition Analysis

Total mineral content (micro- and macroelements) was determined following the AOAC 930.05 procedure [18]. An amount of 0.5 g of the dried sample was incinerated in a microwave oven (Muffle Furnace MLS 1200 model, Monroe, USA). The temperature was increased gradually to 450 °C. The ashes were removed once discolored to a white powder, and were gravimetrically quantified. The extraction of micro-minerals (Fe, Cu, Mn and Zn) from the incinerated residue required an acid mixed of 1 mL HCl (50% v/v) and 1 mL HNO_3_ (50% v/v), and then it was made up to 25 mL with distilled water. These were directly measured by atomic absorption spectroscopy (AAS) through Analyst 200 Perkin Elmer equipment (Waltham, USA) at the adequate and specific wavelength of each element. Standard solutions were carefully prepared for calibration purposes. Regarding macro-minerals (Ca, Mg, K and Na), an additional 1/10 (v/v) dilution was completed in La_2_O_3_ (5.864%, w/v) for the Ca and Mg determination, and in CsCl (1%, w/v) for Na and K analysis. These were also measured by AAS. Total mineral composition was expressed as mg/100 g fw.

## 3. Results and Discussion

### 3.1. Physico-Chemical Parameters

The physico-chemical parameters obtained are summarized in Table 2. The low standard deviation values indicate a low level of mean variance for all of the analyses. Mean values for moisture content, pH and °Brix are quite similar among batches of the same season as well as batches of different seasons. Moisture results ranged between 81.04 and 82.01 g/100 g in batches K1, K2 and K3 (2017 season) and 81.84–83.07 g/100 g in batches K4, K5 and K6 (2018 season). The batches with the highest values of moisture were K3 (82.01 ± 0.40 g/100 g) in the 2017 season and K5 (82.01 ± 0.40 g/100 g) in the 2018 season. All moisture results were higher than the range provided by the official databases of nutritional composition (60–81.4 g/100 g) [29,30,31,32,33,34,35,36]. Moisture values were used to calculate the vitamin C, fiber, mineral and carotenoids contents in persimmon fruit. Regarding pH, mean values varied from 5.84 to 6.34 (2017 season) and from 6.07 to 6.29 (2018 season). Batches K3 and K6 showed the lowest pH results (5.84 ± 0.05 and 6.07 ± 0.07, respectively), whereas batches K1 and K4 had the highest ones (6.34 ± 0.02 and 6.29 ± 0.04, respectively). °Brix mean values for 2017 season showed higher variation between batches (16.00–19.37 °Brix) than 2018 season (15.77–17.10 °Brix). Finally, mean values for titratable acidity were significantly different between seasons (0.4–1.11 meq NaOH (N/100)/100 g in 2017, and 2.87–3.23 meq NaOH (N/100)/100 g in 2018). In the 2017 season, the batch K1 was the one with the lowest values of acidity (0.40 ± 0.00 meq NaOH/100 g) and °Brix (16.00 ± 0.10); however, in 2018 season, the batch with the lowest acidity (K4 = 2.87 ± 0.17 meq NaOH/100 g) showed the highest °Brix result (17.10 ± 0.10).

### 3.2. Fiber Content

Dietary fiber is composed by water-insoluble and water-soluble fiber. Both fractions combined represent the total dietary fiber amount. A diet high in dietary fiber has been shown to be associated with the reduction of the risk of coronary artery disease and diabetes mellitus. Insoluble fiber (cellulose, hemicellulose and lignin) has appeared to improve insulin sensitivity though the precise mechanisms are still unclear. Furthermore, soluble fiber (pectins, gums and mucilages) can lower blood glucose concentration through slowing the absorption of carbohydrates in the gut via increased viscosity [37,38,39,40].

The mean insoluble, soluble and total dietary fiber results are presented in Table 3**,** where values are expressed as g/100 g and g/100 kcal (considering an energy value of 74.5 kcal/100 g), since the requirement for nutritional claims related to fiber in Europe is established both ways. Batches K1, K2 and K3 from 2017 season presented higher insoluble fiber mean results (1.71–2.79 g/100 g fw) than batches K4, K5 and K6 from 2018 season (0.86–1.17 g/100 g fw). Likewise, the soluble fiber mean results of 2017 batches were significantly higher (2.20–3.20 g/100 g fw) than the results of 2018 batches (1.14–1.85 g/100 g fw). Soluble and insoluble fiber fractions are divided approximately into equal parts in these fruits, slightly higher for soluble fiber, compiling the benefits of both fractions. Finally, total dietary fiber mean values for the 2017 batches ranged between 4.47 g/100 g of fw and 4.99 g/100 g of fw, whereas the 2018 batches showed lower values (2.38–2.72 g/100 g fw). On the one hand, the batches with the highest content of total dietary fiber were K2 (4.99 ± 0.26 mg/100 g fw) in 2017 and K6 (2.72 ± 0.19 mg/100 g fw) in 2018. On the other hand, the lowest contents of total dietary fiber were found in the batches K3 (4.47 ± 0.26 mg/100 g fw) and K4 (2.38 ± 0.14 mg/100 g fw) in 2017 and 2018 seasons, respectively. Given that the average weight of persimmon fruits of this experiment is 263.32 g, it can be estimated that just one piece of this fruit can provide between 25.07% and 52.56% of the dietary fiber recommendations (25 g/day) [41]. The great variability in the percentage of the dietary fiber recommendations covered by the consumption of one persimmon fruit is due to the differences found in the dietary fiber content among batches, which directly depends on the nature of the analyzed samples. Biological samples are subject to wide variations because of multiple extrinsic and intrinsic factors of the persimmon fruit such as the environmental and crop conditions and the ripening state during the harvesting process, among others.

The fiber content in persimmon fruits is higher than other more popular fruits and vegetables such as apple (2.0–2.4 g/100 g fw), orange (1.6–2.4 g/100 g fw), pear (2.3–3.2 g/100 g fw), peach (1.6–2.1 g/100 g fw), banana (1.6–3.4 g/100 g fw), spinach (1.9–2.9 g/100 g fw), eggplant (2.4–3 g/100 g fw), zucchini (1.0 g/100 g fw) and green asparagus (1.7–1.8 g/100 g fw) according to the literature [29,30,36].

### 3.3. Vitamin C Content

Vitamin C is an important antioxidant, which plays a key role in metabolism, absorption of iron as well as collagen and L-carnitine biosynthesis, among others. In addition, vitamin C could reduce the risk of atherosclerosis and the development of tumors. Vitamin C deficiency can lead to anemia and scurvy, a serious illness characterized by a strong tendency to bleed as a consequence of disturbances in the collagen metabolism [42,43].

The mean values of AA, DHA and total vitamin C content in 2017 batches are shown in Table 4. The content of DHA (3.56–8.80 mg/100 g fw) was higher than the AA (1.22–1.56 mg/100 g fw) in all batches. This predominance of the oxidized form of vitamin C (DHA) may be explained by a higher oxidative stress that takes place as a response to CO_2_ exposure during deastringent treatment of persimmon fruits, as reported by Novillo et al. (2014) for the same ‘Rojo Brillante’ variety [44]. Batch K3 showed the lowest content of AA (1.22 ± 0.19 mg/100 g fw), DHA (3.56 ± 0.70 mg/100 g fw) and total vitamin C (4.62 ± 0.41 mg/100 g fw) in comparison with batch K1 (1.56 ± 0.49 mg AA/100 g fw, 7.71 ± 0.60 mg DHA/100 g fw and 9.43 ± 1.04 mg total vitamin C/100 g fw) and batch K2 (1.45 ± 0.01 mg AA/100 g fw, 8.80 ± 0.76 mg DHA/100 g fw and 10.25 ± 0.76 mg total vitamin C/100 g fw). Similar results were found in batches K1 and K2, and the content of total vitamin C content of both batches (9.43 ± 1.04 mg/100 g fw and 10.25 ± 0.76 mg/100 g fw, respectively) were in accordance with the values found in the literature (6–70 mg vitamin C/100 g fw) [29,30,31,32,33,34,35,36]. These wide variations in the vitamin C content can be explained by environmental factors, intrinsic characteristics of the fruits, conditions of the crop and time of harvesting. All these factors affect the percentage of the daily reference intake of vitamin C (80 mg/day), which is able to cover the consumption of this fruit. Thus, one piece of persimmon fruit (average weight = 263.32 g) can cover between 15.21% and 33.74% of the daily reference intake of this vitamin [17].

Vitamin C analyses were not carried out in the 2018 batches (K4, K5 and K6) as mean values of the 2017 season were far from meeting the requirements for the authorisation of any nutrition and health claim related to vitamin C in Persimon^®^. Although no values of total vitamin C for ‘Rojo Brillante’ variety are still found in the literature, the ones provided by the DTU Foods Database (FRIDA) (2019), Rizza et al. (2002), Souci et al. (2008), The Spanish Food Composition Database (BEDCA) (2007), and USDA Food Composition Database (2018) agree with the results of the present work in terms of the non-application of nutrition and health-related claims [29,30,34,35,36].

Persimmon fruits contain higher amounts of vitamin C than other more popular fruits and vegetables such as apple (3.0–8.26 g/100 g fw), pear (3.0–6.06 g/100 g fw), plum (3.0–9.5 mg/100 g fw), grapes (3.0–4.0 mg/100 g fw), fig (2.0 mg/100 g fw) and eggplant (2.0–4.0 mg/100 g fw) according to the literature [29,30,36].

The chromatograms obtained for vitamin C analysis in 2017 batches by HPLC-UV-visible are shown in Figure S1, which is provided in the Supplementary Materials.

### 3.4. Carotenoid Profile

Carotenoids are fat-soluble pigments, which can be classified in two main groups according to their chemical composition: carotenes or hydrocarbon compounds (α-carotene, β-carotene, lycopene) and oxycarotenes or xantophylls (lutein, zeaxanthin, β-cryptoxanthin, violaxanthin). The health benefits of carotenoids have been mostly derived from their pro-vitamin A activity in the organism. Vitamin A has been proved to be essential for maintaining a proper health status of the skin, eyes, heart and immune system [38]. α-carotene, β-carotene and β-cryptoxanthin from fruits and vegetables constitute the major source of vitamin A, providing 60% of the total intake of this vitamin. Lycopene, lutein and zeaxanthin cannot be transformed to vitamin A in the organism. However, they play an important role as antioxidants [45,46,47].

In relation to the carotenoid content, interesting results were obtained and are present in Table 5. Chromatograms obtained for carotenoid analysis by HPLC-UV-visible are included in Figure S2, which is provided in the Supplementary Materials. 

A total of five carotenoids were identified and quantified: neoxanthin (up to 4.23 µg/100 g fw), violaxanthin (up to 0.09 µg/100 g fw), β-cryptoxanthin (0.75–3.07 µg/100 g fw), lycopene (17.51–53.50 µg/100 g fw) and β-carotene (10.07–20.50 µg/100 g fw). The total carotenoids content ranged between 35.48 µg/100 g fw and 75.84 µg/100 g fw. In both seasons, the minority carotenoids were neoxanthin, violaxanthin and β-cryptoxanthin. In fact, the neoxanthin could be only quantified in the batches of the 2017 season (K1, K2 and K3) and the violaxanthin in four of the six analysed batches (K1, K2, K3 and K4). The majority carotenoids were the β-carotene and lycopene, with levels of 5 times and 12 times other compounds identified. Similar results were observed by Olives Barba et al. (2006) [26].

The batches of the 2017 season (K1, K2 and K3) contained higher amounts of carotenoids than the 2018 batches (K4, K5 and K6). For instance, the 2017 batches practically doubled the content of violaxanthin (0.04–0.09 µg/100 g fw in 2017 versus not detected –0.03 µg/100 g fw in 2018), β-cryptoxanthin (1.76–3.07 µg/100 g fw in 2017 versus 0.75–1.75 µg/100 g fw in 2018) and β-carotene (10.60–20.50 µg/100 g fw in 2017 versus 10.07 – 13.26 µg/100 g fw in 2018). On the one hand, the batch with the highest content of carotenoids was K2 (4.23 ± 1.92 µg neoxanthin/100 g fw; 0.09 ± 0.01 µg violaxanthin/100 g fw; 3.07 ± 0.55 µg β-cryptoxanthin/100 g fw; 53.50 ± 4.11 µg lycopene/100 g fw, 20.50 ± 3.86 µg β-carotene/100 g fw and 75.84 ± 5.33 µg total carotenoids/100 g fw). On the other hand, batch K5 contained the lowest amounts of carotenoids (neoxanthin and violaxanthin were not detected; 0.75 ± 0.41 µg β-cryptoxanthin/100 g fw; 10.07 ± 2.54 µg β-carotene/100 g fw and 35.48 ± 6.37 µg total carotenoids/100 g fw) with the exception of the lycopene as the batch K4 showed the lowest content of this carotenoid (17.51 ± 6.71 µg lycopene/100 g fw). The differences among batches regarding the vitamin A content expressed as retinol equivalents (RE) is linked to multiple factors (e.g., the ripeness of the fruits by the time of harvesting, environmental conditions, climate and crop, among others) and influence the percentage of the daily reference intake of the vitamin A covered by this fruit. Taking into account that the average weight of one piece is approximately 263.32 g, the consumption of one persimmon fruit can cover between 0.53% and 1.39% of the daily reference intake of this vitamin (800 µg RE/day) [17]. The vitamin A content of the analysed persimmon fruits was higher than other more popular fruits such as lemon (1.0–2.3 µg RE/100 g fw) and lime (0.5–2.0 µg RE/100 g fw) according to the literature [29,30,36].

Giordani et al. (2011), in a review including different varieties of persimmon fruits, reported wide differences in the content of carotenoids, as is expected in every plant material. However, in the aforementioned research only provitamin A carotenoids were studied [48]. The present results showed that the persimmon fruits analysed have low provitamin A activity, while other non-provitamin A carotenoids are mainly present in these fruits. Special mention should be made for the lycopene, a non-provitamin A carotenoid widely studied due to its high antioxidant potential. Novillo et al. (2015) found levels of β-carotene in ‘Rojo Brillante’ persimmon fruits similar to those found in this study; and higher xantophylls contents; however, these authors did not report lycopene levels [8]. On the other hand, Plaza et al. (2012) showed that lycopene is the carotenoid which experiments a higher increase during the ripeness of ‘Rojo Brillante’ persimmon fruits (9–43 µg/100 g), while for other carotenoids, the content remains similar during the ripening process [11]. This content is in agreement with the red color of ‘Rojo Brillante’ persimmon fruits. Lycopene is an interesting carotenoid due to its biological activity as an antioxidant with different beneficial health effects. Since there are not many dietary sources of lycopene, apart from tomato, watermelon, pink grapefruit or guava, ‘Rojo Brillante’ persimmon fruits, with 26.76–53.5 µg/100 g fw, may contribute to lycopene intake in the daily diet.

It is interesting that in a recent review including different varieties of persimmon fruits, it was found that β-carotene was absent in most astringent varieties [49]. This fact could explain the low concentrations of β-carotene found in ‘Rojo Brillante’ persimmon fruits. On the other hand, opposite results were found in another study carried out with 46 different persimmon cultivars (32 astringent and 14 non-astringent) as the astringent varieties showed significant β-carotene amounts [50]. Therefore, a high variability exists in these parameters, in agreement with the results of the present work.

### 3.5. Mineral Composition

Mineral composition of food matrices has a vital role in human health as well. Not only can the lack of certain minerals lead to deficiencies, but also they have been suggested to be effective in reducing the risk of specific diseases. Examples include magnesium in the prevention of atherosclerosis and potassium in reducing the risk of hypertension [51]. Furthermore, certain macro (Ca, Mg, K, Na) and micro-minerals (Fe, Cu, Zn, Mn) are required and sometimes essential for enzymatic reactions such as calcium for muscle contraction and blood clotting [52,53]. The mineral composition of the six analysed batches of Persimon^®^ is shown in Table 6. Mean values of micro- (Fe, Cu, Zn, Mn) and macro-minerals (Ca, Mg, Na, K) are generally similar among batches of the same season but not between seasons. Microminerals mean values of 2018 batches (K4, K5 and K6) were higher than the 2017 season, contrary to macrominerals mean values, which were lower than batches K1, K2 and K3. With the exception of the Mn and Na contents, the batch K3 showed the highest micro- and macro-minerals values in 2017 season (0.23 ± 0.00 mg Fe/100 g fw; 0.28 ± 0.00 mg Cu/100 g fw; 0.69 ± 0.07 mg Zn/100 g fw; 36.63 ± 2.39 mg Ca/100 g fw; 20.95 ± 1.49 mg Mg/100 g fw and 349.11 ± 1.46 mg K/100 g fw), whereas the batch K1 had the lowest contents of the analysed minerals without counting the Mn and Ca (0.15 ± 0.00 mg Fe/100 g fw; 0.26 ± 0.01 mg Cu/100 fw; 0.11 ± 0.01 mg Zn/100 g fw; 13.65 ± 0.38 mg Mg/100 g fw; 3.84 ± 0.12 mg Na/100 g fw and 251.24 ± 3.20 mg K/100 g fw). More similar values were found among batches of the 2018 season. The batch K4 showed the highest content of Cu (3.93 ± 0.17 mg/100 g fw), Zn (0.35 ± 0.03 mg/100 g fw), Ca (12.56 ± 0.21 mg/100 g fw), Mg (7.78 ± 0.76 mg/100 g fw) and Na (1.92 ± 0.07 mg/100 g fw). The lowest values of Mn (0.23 ± 0.02 mg/100 g fw), Ca (10.36 ± 0.33 mg/100 g fw), Mg (7.03 ± 0.12 mg/100 g fw) and Na (1.61 ± 0.08 mg/100 g fw) were found in the batch K5. As previously mentioned, biological samples like the analyzed persimmon fruits, are subject to wide variations regarding chemical composition due to either extrinsic factors (environmental and crop conditions) or intrinsic characteristics of the fruit. In this respect, mineral elements are probably the parameters presenting wider variations among batches, which are reflected in the percentage of the daily reference intake of minerals covered by this fruit. As the weight of one piece of persimmon fruit is approximately 263.32 g, its consumption can contribute to achieve the daily reference intakes of the following minerals: 2.8%–78.4% (Fe), 2.9%–18.2% (Zn), 7.9%–34.2% (Mn), 3.4%–12.1% (Ca), 4.9%–14.7% (Mg) and 13.3%–46.0% (K) [17].

Mean values for Mn and Na in both seasons are in accordance with the reported values (0–0.355 mg Mn/100 g fw and 1–10 mg Na/100 g fw), whereas the content of Cu and Zn in all analysed batches was higher than those found in the literature (0.02–0.113 mg Cu/100 g fw and 0–0.110 mg Zn/100 g fw) [29,30,31,32,33,34,35,36]. Regarding Mg and K content, mean values of 2018 batches fell into the reported range (6–11 mg Mg/100 g fw and 135–208 mg K/100 g fw), and Mg and K amounts in the 2017 batches (K1, K2 and K3) were higher than in literature. Conversely, Fe content in the 2017 batches was within the reported level of 0–0.4 mg Fe/100 g fw while the mean values of the 2018 season were higher. Finally, Ca amounts found in all analyzed batches (excluding K3) were supported by the values provided by the aforementioned authors and databases (7–21 mg Ca/100 g fw).

The content of some micro- and macro-minerals analyzed in the persimmon fruits was higher than other more popular fruits and vegetables such as apple (0.02–0.10 mg Zn/100 g fw; 4.13–6.00 mg Ca/100 g fw and 4.49–5.00 mg Mg/100 g fw), pear (6.46–7.00 mg Mg/100 g fw), banana (5.00–9.00 mg Ca/100 g fw), mango (0.08–0.10 mg Zn/100 g fw), peach (0.06–0.17 mg Zn/100 g fw and 6.00–8.00 mg Ca/100 g fw), blueberry (77.00–103.00 mg K/100 g fw), artichoke (0.10–0.12 mg Zn/100 g fw) and eggplant (8.40–10.00 mg Ca/100 g fw) according to the literature. Regarding the content of Na, the analyzed persimmon fruits showed lower amounts of this mineral than other vegetables like eggplant (7.00–15.00 mg Na/100 g fw) and artichoke (4.00–27.00 mg Na/100 g fw) [29,30,36].

From all the results presented, it can be concluded that the fruit harvest in the 2018 season had, in general, higher mineral contents compared to those of the 2017 season; while for other components such as fiber and vitamins the contrary trend was found (2017 higher than 2018), being fruits from batch K2 richer in fiber, vitamin C and carotenoids (with more β-carotene, lycopene, RE, and the presence of special compounds such as violaxanthin and neoxanthin, which are not always present in all the fruits).

### 3.6. Potential Nutrition Claims Applied to Deastringed ‘Rojo Brillante’ Persimmon Fruits (Diospyros kaki L.), Protected Designation of Origin (PDO) ‘Ribera del Xúquer’

Persimon^®^ fruit must contain at least 3 g of total dietary fiber per 100 g of edible portion or at least 1.5 g per 100 kcal for the application of the nutrition claim “Source of fiber”. As the total dietary fiber mean values of all analysed batches (2017 and 2018 seasons) are higher than the requirement of 1.5 g of fiber/100 kcal, it would be possible to make the nutrition claim “Source of fiber” in Persimon^®^ fruits. Regarding the nutrition claims related to vitamins and/or minerals “Source of [name of vitamin/s] and/or [name of mineral/s]”, the significant amount required for making these claims corresponds to 15% of Nutrient Reference Values (NRV), as supplied by 100 g of food. The nutrition claim “Sodium-free or salt-free” could be applied to Persimon^®^ fruit as its content in sodium is lower than the requirement of 0.005 g of sodium. The nutrition claims “Source of vitamin C” and “Source of manganese” are not applicable to these fruits as Persimon^®^ fruit does not contain more than 12 mg vitamin C/100 g and 0.3 mg Mn/100 g. Likewise, it is not possible to make the nutrition claim “Source of iron” because the persimmon fruit only met the specific requirement (mean values >2.1 mg Fe/100 g) in the 2018 season but not in 2017 [14]. Regarding the micromineral Cu, its mean values of all analyzed batches (2017 and 2018 seasons) were higher than the aforementioned significant amount (0.15 mg Cu/100 g of the product), indicating that Persimon^®^ fruit could a priori be a potential “Source of copper”. However, some limitations should be taken into account regarding this nutrition claim since copper salts are approved products usually used in many crops (even in ecological agricultural practices) in order to fight against some pests. Therefore, copper content in the analyzed persimmons may not be intrinsic to these fruits but due to agricultural practices applied. The same consideration can be applied to health claims, according to the Annex of Regulation (EC) No 432/2012, due to the Cu content [54]. In the authors’ opinion, no health-related claims regarding Cu can be safely applied and more studies could be useful for supporting this finding.

## 4. Conclusions

Persimmon fruit contains bioactive compounds such as fiber, vitamin C, carotenoids as well as micro- and macro-minerals with important health-promoting effects. The utilization of persimmon fruit and its bioactive components may be a good strategy to improve the health status of the population. In the present work, ‘Rojo Brillante’ persimmon fruits (*Diospyros kaki* L.), PDO ‘Ribera del Xúquer’, have been characterized regarding the presence of fiber with a total content of 2.38–4.99 g/100 g fw (slightly higher for soluble fiber); vitamin C in the range of 4.62–10.25 g/100 g fw (mainly in the form of dehydroascorbic acid); carotenoids (with lycopene as the predominant one, in 26.76 – 51.10 µg/100g fw, followed by β-carotene, in 10.07–20.50 µg/100g fw, and neoxanthin, violaxanthin and β-cryptoxanthin as minor compounds); and mineral elements (Fe, Cu, Zn, Mn, Ca, Mg, Na and K). Based on the data in this work, Persimon^®^ fruit is potentially able to show two nutrition claims: “Source of fiber” and “Sodium-free or salt-free”. This work could set the ground for future studies on this or other persimmon varieties, as well as for starting to consider natural food products as candidates for the use of approved nutrition claims.

## Figures and Tables

**Table 1:** Solubilization conditions, linearity and sensitivity parameters for carotenoid compounds analysed.

Compound	Solvent Stock Solutions	Solvent Work Solution	RT (min)	Range of Linearity (µg/mL)	Correlation Coefficient (R^2^)	LD	LQ
Neoxanthin	Ethyl eter	ACN	5.57	2.5–20	0.9915	2.60	8.69
Violaxanthin	Ethyl eter	ACN	6.15	0.1–5	0.9955	0.51	1.70
β-Cryptoxanthin	Cloroformo	Hexane	15.69	1–15	0.9992	0.15	0.52
Lycopene	THF:ACN:MetOH (15:30:55, v:v:v)	Hexane	19.14	2–20	0.9969	0.83	2.76
β-Carotene	Cloroformo	Hexane	28.57	0.25–5	0.9984	0.15	0.509

RT: retention time; LD: limit of detection; LQ: limit quantification; ACN: acetonitrile.

**Table 2:** Moisture, pH, acidity and ºBrix values of deastringed persimmon fruits (‘Rojo Brillante’ variety) were analysed in the 2017 and 2018 seasons. Results expressed as mean ± standard deviation (*n*-1), *n* = 3. Average weight of persimmon fruits = 263.32 g.

Batch	Moisture (g/100 g)	pH	Acidity (meq NaOH/100 g)	°Brix
*2017 season*				
K1	81.04 ± 0.08	6.34 ± 0.02	0.40 ± 0.00	16.00 ± 0.10
K2	81.47 ± 0.10	6.27 ± 0.04	1.11 ± 0.01	19.37 ± 0.12
K3	82.01 ± 0.40	5.84 ± 0.05	0.78 ± 0.03	18.73 ± 0.12
*2018 season*				
K4	81.84 ± 0.02	6.29 ± 0.04	2.87 ± 0.17	17.10 ± 0.10
K5	83.07 ± 0.05	6.24 ± 0.08	2.94 ± 0.17	15.77 ± 0.06
K6	82.23 ± 0.07	6.07 ± 0.07	3.23 ± 0.29	16.57 ± 0.12
**Range**	81.04–83.07	5.84–6.34	0.40–3.23	15.77–19.37

**Table 3:** Insoluble, soluble and total dietary fiber values (mg/100g fw) of deastringed persimmon fruits (‘Rojo Brillante’ variety) analysed in the 2017 and 2018 seasons. Results expressed as mean ± standard deviation (*n*-1), *n* = 3.

Batch	Insoluble Fiber (mg/100 g)	Soluble Fiber (mg/100 g)	Total Dietary Fiber (mg/100 g)	Total Dietary Fiber (mg/100 kcal)
*2017 season*				
K1	1.71 ± 0.13	3.20 ± 0.19	4.90 ± 0.34	6.57 ± 0.46
K2	2.79 ± 0.26	2.20 ± 0.15	4.99 ± 0.26	6.69 ± 0.35
K3	2.60 ± 0.17	2.31 ± 0.17	4.47 ± 0.26	5.99 ± 0.26
*2018 season*				
K4	1.17 ± 0.02	1.14 ± 0.06	2.38 ± 0.14	3.19 ± 0.19
K5	0.89 ± 0.03	1.64 ± 0.12	2.54 ± 0.10	3.41 ± 0.13
K6	0.86 ± 0.02	1.85 ± 0.18	2.72 ± 0.19	3.65 ± 0.25
**Range**	0.86–2.79	1.14–3.20	2.38–4.99	3.19–6.69

**Table 4:** Vitamin C values (mg/100g fw) of deastringed persimmon fruits (‘Rojo Brillante’ variety) analysed in the 2017 season. Results expressed as mean ± standard deviation (*n*-1), *n* = 3.

Batch	Ascorbic Acid (AA) (mg/100 g)	Dehydroascorbic Acid (DHA) (mg/100 g)	Total Vitamin C (mg/100 g)
K1	1.56 ± 0.49	7.71 ± 0.60	9.43 ± 1.04
K2	1.45 ± 0.01	8.80 ± 0.76	10.25 ± 0.76
K3	1.22 ± 0.19	3.56 ± 0.70	4.62 ± 0.41
**Range**	1.22–1.56	3.56–8.80	4.62–10.25

**Table 5:** Carotenoids content (µg/100g fw) of deastringed persimmon fruits (‘Rojo Brillante’ variety) analysed in the 2017 and 2018 seasons. Results are expressed as mean ± standard deviation (*n*–1), *n* = 183.

Batch	Neoxanthin (µg/100 g)	Violaxanthin (µg/100 g)	β-Cryptoxanthin (µg/100 g)	Lycopene (µg/100 g)	β-Carotene (µg/100 g)	Total Carotenoids (µg/100 g)	RE (µg/100 g)
		*2017 season*					
K1	3.18 ± 0.25	0.06 ± 0.02	1.76 ± 0.41	26.60 ± 3.40	10.60 ± 0.70	40.86 ± 4.31	3.84 ± 0.12
K2	4.23 ± 1.92	0.09 ± 0.01	3.07 ± 0.55	53.50 ± 4.11	20.50 ± 3.86	75.84 ± 5.33	4.21 ± 0.06
K3	1.08 ± 0.34	0.04 ± 0.04	2.77 ± 0.53	42.20 ± 1.31	15.55 ± 0.62	60.24 ± 1.54	3.97 ± 0.00
		*2018 season*					
K4	nd	0.03 ± 0.01	1.75 ± 0.62	17.51 ± 6.71	12.38 ± 3.44	47.46 ± 10.12	1.92 ± 0.07
K5	nd	nd	0.75 ± 0.41	26.74 ± 3.43	10.07 ± 2.54	35.48 ± 6.37	1.61 ± 0.08
K6	nd	nd	1.32 ± 0.40	41.97 ± 5.12	13.26 ± 5.12	41.38 ± 10.61	1.66 ± 0.13
**Range**	nd–4.23	nd–0.09	0.75–3.07	17.51–53.50	10.07–20.50	35.48–75.84	1.61–4.21

RE = retinol equivalents.

**Table 6:** Mineral content (mg/100g fw) of deastringed persimmon fruits (‘Rojo Brillante’ variety) analysed in 2017 and 2018 seasons. Results expressed as mean ± standard deviation (*n*–1), *n* = 183.

Batch	Fe (mg/100 g)	Cu (mg/100 g)	Zn (mg/100 g)	Mn (mg/100 g)	Ca (mg/100 g)	Mg (mg/100 g)	Na (mg/100 g)	K (mg/100 g)
		*2017 season*						
K1	0.15 ± 0.00	0.26 ± 0.01	0.11 ± 0.01	0.07 ± 0.01	14.41 ± 0.48	13.65 ± 0.38	3.84 ± 0.12	251.24 ± 3.20
K2	0.19 ± 0.02	0.28 ± 0.03	0.26 ± 0.01	0.06 ± 0.01	14.17 ± 0.51	19.50 ± 0.96	4.21 ± 0.06	279.67 ± 24.43
K3	0.23 ± 0.00	0.28 ± 0.00	0.69 ± 0.07	0.06 ± 0.01	36.63 ± 2.39	20.95 ± 1.49	3.97 ± 0.00	349.11 ± 1.46
		*2018 season*						
K4	4.12 ± 0.33	3.93 ± 0.17	0.35 ± 0.03	0.25 ± 0.00	12.56 ± 0.21	7.78 ± 0.76	1.92 ± 0.07	101.30 ± 10.10
K5	4.13 ± 0.33	3.36 ± 0.02	0.35 ± 0.03	0.23 ± 0.02	10.36 ± 0.33	7.03 ± 0.12	1.61 ± 0.08	104.00 ± 8.76
K6	4.17 ± 0.39	3.33 ± 0.29	0.30 ± 0.03	0.26 ± 0.02	11.09 ± 1.04	7.78 ± 0.55	1.66 ± 0.13	104.36 ± 10.40
**Range**	0.15–4.17	0.26–3.93	0.11–0.69	0.06–0.26	10.36–36.63	7.03–20.95	1.61–4.21	101.30–349.11

## Supplementary Materials

The following are available online: https://www.mdpi.com/2072-6643/12/5/1397/s1. Figure S1. Chromatograms obtained for vitamin C analysis by HPLC-UV-visible: (a) commercial standard of ascorbic acid; (b) extract of deastringed persimmon fruit ‘Rojo Brillante’ PDO ‘Ribera del Xúquer’; (c) Extract of deastringed persimmon fruit ‘Rojo Brillante’ PDO ‘Ribera del Xúquer’, after reduction with L-cystein. Chromatographic conditions: see text. Figure S2. Chromatograms obtained for carotenoid analysis by HPLC-UV-visible: (a) commercial standards; (b) extract of deastringed persimmon fruit ‘Rojo Brillante’ PDO ‘Ribera del Xúquer’. Chromatographic conditions: see text.
