# Supplementary material for: Potential Nutrition and Health Claims in Deastringed Persimmon Fruits (Diospyros kaki L.), Variety ‘Rojo Brillante’, PDO ’Ribera del Xúquer’
Source: Nutrients. 2020 May 13;12(5):1397. doi: 10.3390/nu12051397 (PMC7284415; doi:10.3390/nu12051397)
Supplement: Supplementary file 1 [file nutrients-12-01397-s001.pdf]

Figure S2. Chromatograms obtained for vitamin C analysis by HPLC-UV-visible: (**a**) Commercial standard of ascorbic acid; (**b**) Extract of deastringed persimmon fruit 'Rojo Brillante' PDO 'Ribera del Xúquer'; (**c**) Extract of deastringed persimmon fruit 'Rojo Brillante' PDO 'Ribera del Xúquer', after reduction with L-cystein. Chromatographic conditions: see text.

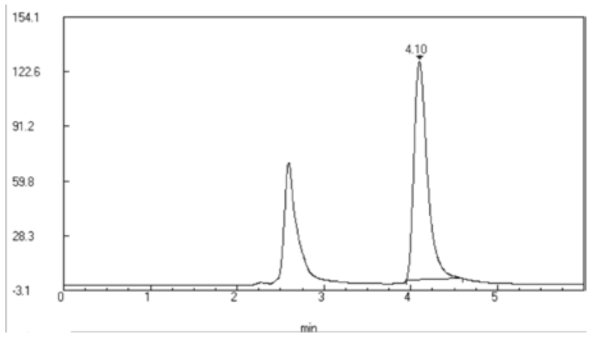

(**a**)

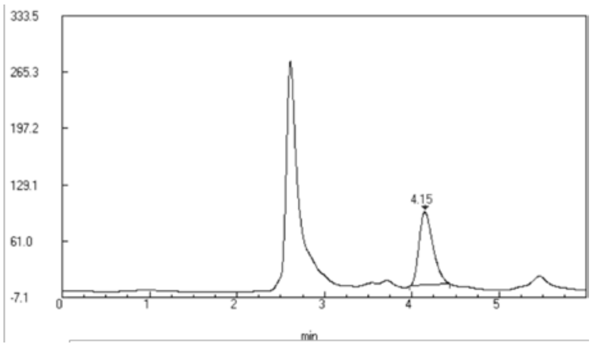

(**b**)

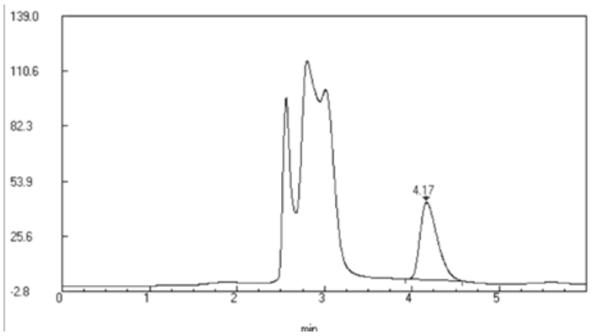

(**c**)

Figure S3. Chromatograms obtained for carotenoid analysis by HPLC-UV-visible: (**a**) Commercial standards; (**b**) Extract of deastringed persimmon fruit 'Rojo Brillante' PDO 'Ribera del Xúquer'. Chromatographic conditions: see text.

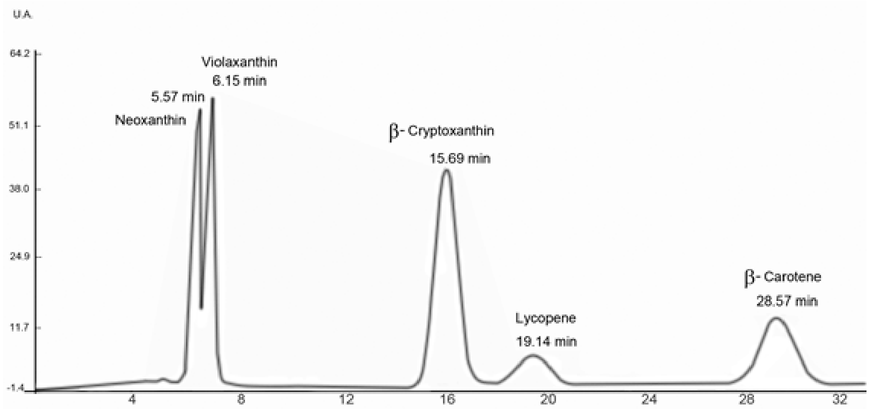

(**a**)

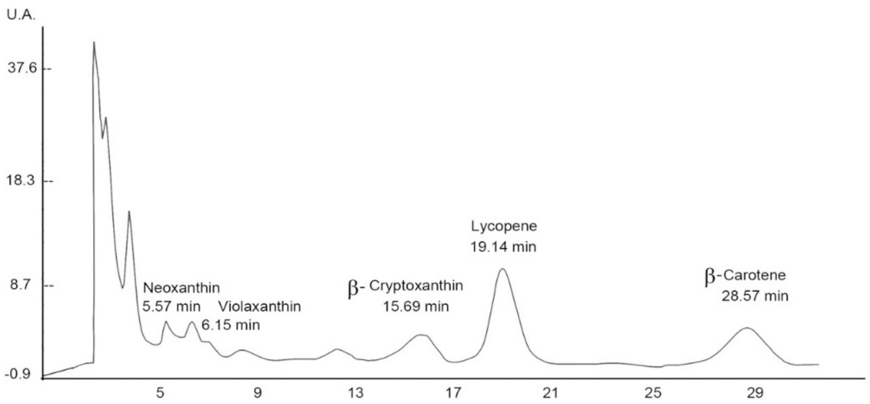

(**b**)
